# Long noncoding RNA *ENST00000508435* promotes migration of breast cancer via FXR 1

**DOI:** 10.1080/19336918.2021.1921402

**Published:** 2021-05-30

**Authors:** Luying Li, Youping Jin, Xue Wang, Li Wang, Yangbai Sun, Yihong Luo, Xiaojian Ni, Qi Lu, Wenbo Wei, Xiuling Zhi, Jerry Yu, Wei Zhu, Ping Zhou

**Affiliations:** a Department of Physiology and Pathophysiology, School of Basic Medical Sciences, Fudan University, Shanghai, China; b Institutes of Biomedical Sciences, Fudan University, Shanghai, China; c Pudong Hospital, Fudan University, Shanghai, China; d Department of Musculoskeletal Oncology, Fudan University Shanghai Cancer Center, Shanghai Medical College, Fudan University, Shanghai, China; e Department of General Surgery, Zhongshan Hospital, Fudan University, Shanghai, China; f Department of Medicine, University of Louisville, Louisville, KY, USA

**Keywords:** Breast cancer, lncRNA *ENST00000508435*, FXR1, migration, metastasis

## Abstract

LncRNA plays a critical role in tumor progression. However, the role it executes in breast cancer is still unclear. Here, we report a newly discovered lncRNA, *ENST00000508435*, which could be remarkably up-regulated in breast cancer cells and tissues. We found that the expression of *ENST00000508435* was positively correlated with tumor size, lymph node metastasis and HER2. More interesting, overexpression of *ENST00000508435* significantly increased cell migration, while specific knockdown led to the opposite. RNA pull-down and RNA immunoprecipitation assays demonstrated that *ENST00000508435* could directly bind to FXR1 to promote tumor metastasis. *ENST00000508435* and FXR1 were positively correlated. FXR1 was also significantly up-regulated in breast tumors. Taken together, we propose that *ENST00000508435* regulates FXR1 to promote breast cancer metastasis.

## Introduction

Breast cancer, with high metastasis and lethal characteristics, has lately been reported to surpass lung cancer as the most commonly diagnosed cancer, with an estimated 2.3 million new cases (11.7%), followed by lung cancer (11.4%) in the world [1]. Although its clinical intervention has been improved in recent years, the prognosis still remains unsatisfactory, and molecular mechanisms of its metastasis remain unrevealed. Therefore, there is an urgent need to determine its underlying mechanisms to provide novel targets for prevention and/or treatment of breast cancer.

The human genome can express more than 100,000 proteins via transcription and translation. Only 2% of our genomic transcripts can encode proteins. The rests are noncoding RNAs (ncRNAs). The ncRNAs can be divided into two types according to RNA length: small RNA (<200bp) and long non-coding RNAs (lncRNAs) (>200bp) [2]. Based on the genetic location and transcriptional direction, lncRNAs can be further divided into: sense lncRNAs, antisense lncRNAs, intergenic lncRNAs and divergent lncRNAs [3]. Most lncRNAs are transcribed by RNA polymerase II, with 5′ methyl cap and 3′ polynucleotide tail [3]. Reportedly, lncRNAs interact with DNAs, proteins and other RNAs, and participate in the processes of transcriptional, post transcriptional and epigenetic levels [4]. LncRNAs are responsible for tumorigenesis, and play pivotal roles in tumor cell proliferation, apoptosis, migration and invasion [4]. Using microarray, we previously identified a number of lncRNAs that were aberrantly expressed in breast cancer tissues [5]. Nevertheless, the expression and function of lncRNA *ENST00000508435* in breast cancer have not been studied intensively.

In the present study, we hypothesize that ectopic lncRNA *ENST00000508435* involves breast cancer progression. To begin with, quantitative real-time PCR was executed to validate the expression of lncRNA *ENST00000508435* in breast cancer cell lines and tumor tissues. We found that it was significantly higher in both breast tumor cells and tissues than those in normal breast epithelial cells and peritumor tissues, respectively. Together with some experimental results, we figured out that the ectopic *ENST00000508435* was associated with tumor size, lymph node metastasis and HER2 in breast cancer patients. In addition, we used RNA pull-down assay, RNA immunoprecipitation (RIP) to explore the target protein of *ENST00000508435* in the metastasis. At last, we performed *in vivo* experiments to confirm the function of *ENST00000508435* in breast cancer. All in all, our results support an important role of *ENST00000508435* in breast cancer metastasis.

## Materials and methods

### Tumor tissues and cell lines

This study was carried out in accordance with the World Medical Association Declaration of Helsinki and approved by the Basic Medical Ethics Committee of Fudan University (2017-F001). All 127 pairs of fresh breast cancer tissues and matched paracancerous tissues were collected from Zhongshan Hospital, Fudan University. The hematoxylin–eosin-stained frozen sections contained more than 70% of sufficient epithelial elements. The human breast cancer cell lines MDA-MB-231 (RRID: CVCL_0062), BT549 (RRID: CVCL_1092), MCF-7 (RRID: CVCL_0031) and T47D (RRID: CVCL_0553) were obtained from Shanghai Type Culture Collection of the Chinese Academy of Sciences. Human mammary epithelial cell lines MCF10A (RRID: CVCL_0598) were purchased from the American Type Culture Collection. Human mammary epithelial cell lines MCF10A were purchased from the American Type Culture Collection. BT549 and T47D were routinely cultured in RPMI-1640 medium supplemented with 10% FBS (Gibco, Grand Island, NY). MDA-MB-231 and MDA-MB-468 were cultured in L-15 medium (Gibco, Grand Island, NY, Grand Island, NY) with 10% FBS without CO_2_. MCF-7 cell lines were maintained in DMEM (high glucose) (Gibco, Grand Island, NY) supplemented with 10% FBS. MCF10A cell lines were maintained in F12 medium (Gibco, Grand Island, NY) supplemented with insulin (10 mg/mL; Sigma), EGF (100 mg/mL; Sigma), hydrocortisone (1 mg/mL; Sigma), choleric toxin (100 ng/mL; Sigma), and 5% horse serum (Gibco, Grand Island, NY). All cells were supplemented with 100 U/mL penicillin G and 100 μg/mL streptomycin (Gibco, Grand Island, NY).

### Real-time quantitative PCR assay

Total RNA for cell lines was harvested with Trizol (Life Technologies, Rockville, MD) according to the manufacturer’s instructions. RNA was reversely transcribed to cDNA using PrimeScript RT reagent kit (Toyobo, Japan). The cDNA was amplified with a CFX96 real-time PCR system (Bio-Rad, Hercules, CA) using SYBR Green PCR Master Mix (Bio-Rad) to determine the transcription level of specific genes. GAPDH was used for normalization. The expression level of objective RNA was calculated by the 2^−ΔΔCt^ method. Primer sequences applied were as follows: *GAPDH*, (F: 5′-GGG AAA CTG TGG CGT GAT-3′, R: 5′-GAG TGG GTG TCG CTG TTG A-3′); *ENST00000508435-1*, (F: 5′-GAG AAG GAT AAG CAC ACT G-3′, R: 5′-ATC AAC AGG ACC TCT GGA T-3′); *ENST00000508435-2*, (F: 5′-ATC AAT TCT ATA ATT CCC TTT CCC CTC-3′, R: 5′-AAC CAC TTA TTT CTC CAT CCT TTG C-3′); *FXR1*, (F: 5′-ACG AGC TGA GTG ATT GGT CA −3′, R: 5′-CTG TGA TGA GAT TCG CTG GC-3′).

### Western blot

Cells were washed twice in cold phosphate-buffered saline solution (PBS) and lysed in ice-cold RIPA buffer containing protease inhibitors and phosphatase inhibitors. Equal amounts of proteins were separated with 6%–12% SDS-PAGE gel and blotted onto poly-vinylidene fluoride membranes. The membranes were blocked 1–2 h at room temperature with 5% skim milk and incubated with primary antibodies against FXR1 (Abcam Cat# ab56386, RRID: AB_2110688) and β-actin (Santa Cruz Biotechnology Cat# sc-47,778, RRID: AB_626632) at 4°C overnight, followed by PBS Tween wash. The samples were incubated with secondary antibodies for 1–2 h at room temperature, and protein bands were then visualized with Quantity One software (Bio-Rad). The expression level was normalized with β-actin.

### Plasmid and siRNA transfection

The full length of cDNA encoding *ENST00000508435* was PCR-amplified with primers and inserted into the BamHI and XhoI sites of the pcDNA3.1 vector named pcDNA-*ENST00000508435*. The siRNAs to target *ENST00000508435*, FXR1, and nonspecific controls were constructed by GenPharma (Shanghai, China). Transfections were performed with Lipofectamine 2000 (Invitrogen, CA) in Opti-MEM (Gibco, Grand Island, NY) according to the manufacturer’s instructions. Cells were harvested after 48 h. The siRNA sequences follows: ENST siRNA-homo-358 (sense 5′-GCC AGA GAA GGA UAA GCA CTT-3′, antisense 5′-GCC AGA GAA GGA UAA GCA CTT-3′); ENST siRNA-homo-317 (sense 5′- GAG AAU AGA GAG GCA CAA ATT-3′, antisense 5′- UUU GUG CCU CUC UAU UCU CTT-3′); ENST siRNA-homo-215 (sense 5′-GCA AAG GAU GGA GAA AUA ATT-3′, antisense 5′-UUA UUU CUC CAU CCU UUG CTT-3′); and FXR1 siRNA (sense 5′-GCA AAU GAC CAA GAG CCA UTT-3′, antisense 5′-AUG GCU CUU GGU CAU UUG CTT-3′).

### Cell fractionation assay

The cell fractionation assay was performed by the Paris Kit (life, Part Number AM1921, Grand Island, NY) according to the manufacturer’s instructions. In brief, up to 10^7^ fresh cultured cells, resuspended in 300 to 500 μL ice-cold cell fractionation buffer, and incubated on ice for 5 to 10 min; after centrifugation, the nuclear pellet was lysed in cell disruption buffer, mixed with 2× lysis/binding solution and ethanol, and washed; RNA was then eluted.

### Transwell migration assay

To test cells’ migratory ability, transwell migration experiment was performed in 8-μm-pore transwell inserts (Corning Costar, Cambridge, MA). Therein, 0.5 × 10^5^ cells were seeded in the upper chamber in serum-free medium. Add medium containing 10% FBS as a chemoattractant to the lower chamber. After incubating for 24 h, wiped the cells that failed to migrate through the pores with a cotton swab, fixed the filter membrane with 4% paraformaldehyde, and stained with 0.1% crystal violet. Cell numbers were counted and analyzed in six random fields per well.

### Wound healing assay

pcDNA-*ENST00000508435* and siRNA were transiently transfected into MDA-MB-231 and BT549 cells in 6-well plates. After 48 h, when the cells at approximately 90% confluence, scraped the cell monolayer with a 200-μL pipette tip to create a wound gap. The wounded monolayers were then washed with PBS and cultured for various amounts of time. Five fields were randomly selected in each scratch wound, and the scratches were photographed microscopically. The gap lengths were measured with Image J software. Overexpression of *ENST00000508435* in the MDA-MB-231 cells and the scraped cells was monitored by a JulI Smart fluorescent cell analyzer. The movement of cells was observed in the same field of view, and photographs were taken every 20 min for 36 h.

### RNA pull-down

LncRNA ENST00000508435 or antisense lncRNA ENST00000508435 were transcribed in vitro from vector pcDNA3.1 and biotin-labeled T7 RNA polymerase (Roche, Basel, Switzerland) using Biotin RNA Labeling Mix (Roche). Biotinylated RNAs were dissolved with RNase-free DNaseI (Roche) and purified with an RNeasy Mini Kit (Qiagen, Valencia, CA). Whole-cell lysates (1 mg) from BT549 cells were then incubated with 3 μg of purified biotinylated transcripts at 4°C for 1 h. Washed streptavidin agarose beads (Invitrogen) were added to the protein-RNA complexes and incubated at room temperature for 1 h, washed three times, and boiled at 95°C in SDS buffer. The protein was then detected via SDS gel electrophoresis.

### RNA immunoprecipitation

RIP was performed with the Magna RIP RNA-binding protein immunoprecipitation kit (Millipore, Bedford, MA) and FXR1 (CST, Beverly, MA) according to the manufacturer’s instructions. In brief, beads were mixed with IgG or FXR1 antibody and cell lysate, rotated at room temperature for 4 h, and washed five times. The co-precipitated RNAs were detected via RT-qPCR.

### Microfluidic chip invasion assay

A microfluidic device with various microchannels was used to mimic the invasion and migration process in vivo. The system comprised a PDMS (polydimethylsiloxane; Corning, NY)–glass device connected with a syringe pump and a Petri dish. It contained several micro channels for functions such as culture medium supply, cell seeding, and Matrigel (Corning) loading. Briefly, cells were seeded into the cell culture channel of the microfluidic chip at a density of 5 × 10^4^ cells/cm^2^ with a medium containing 10% FBS. The chip was then turned on its side to allow the cells to adhere to the surface of the gel. The cells were allowed to invade for 12 h to evaluate their invasion abilities, and DAPI was applied to stain the nuclei.

### Animal studies

MDA-MB-231 cells (2 × 10^6^) with *ENST00000508435* overexpression and control vectors were respectively resuspended in 100 μL of PBS, and then were injected into the tail veins of 8-week-old female BALB/c nude mice (eight mice in each group) to generate metastasis, respectively. Four weeks later, the mice were euthanatized with an overdose of anesthetic. The mice were first anesthetized via subcutaneous injection of pentobarbital sodium. The initial dose given to mice was 65 mg/kg, and an additional 3-mg dose would be given according to actual experimental needed. The metastatic burden in the lungs was quantified from four hematoxylin–eosin-stained sections. All animal experiments were performed in the Department of Physiology and Pathophysiology, School of Basic Medical Sciences, Fudan University. Related animal studies were followed National Institutes of Health guide for the care and use of Laboratory animals. The protocol and procedures employed were ethically reviewed and approved by the Institutional Animal Care and Use Committee at Fudan University (20160927–4).

### Statistical analysis

Data were analyzed with SPSS 17.0 (Chicago, IL, USA). Two-tailed student^’^s t-tests or one-way ANOVA were used. A *P* value of less than 0.05 was considered for a significant influence. The diagrams were processed with Prism 6.0 (GraphPad Software, La Jolla, CA, USA).

## Results

### Expression of *ENST00000508435* increases in human breast cancer tissues and cell lines

**Figure 1.**
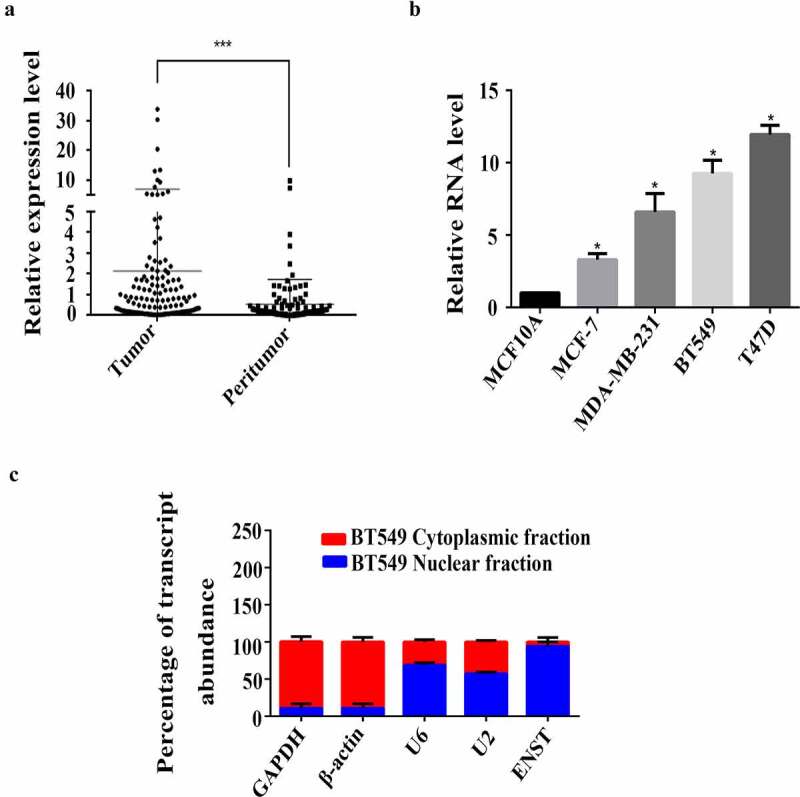
Significant up-regulation of lncRNA *ENST00000508435* (ENST for short) in breast cancer tissues and cell lines. (a) RT-qPCR detection in 127 pairs of fresh tissues from the breast cancer patients showed that lncRNA *ENST00000508435* was highly expressed in breast cancer tissues. ΔΔCt = ΔCt (cancer)-ΔCt (normal), ΔCt = Ct (lncRNA *ENST00000508435*)-Ct (GAPDH), ***P< 0.001 versus control. (b) RT-qPCR analysis of the expression of lncRNA *ENST00000508435* in four different breast cancer cell lines (n = 4, mean ±S.D.) *P< 0.05 versus control. (c) Fractionation of BT549 cells followed by RT-qPCR. lncRNA *ENST00000508435* is mainly located in the cell nucleus. GAPDH and β-actin are acted as internal references for cytoplasmic distribution. U6 and U2 are defined as the references of the cell nucleus

We previously reported that in breast cancer tissues, 224 lncRNAs were upregulated and 324 lncRNAs were downregulated compared to para-carcinoma tissues (NCBI GEO accession: GSE80266) [5]. Further analyses of our previously constructed microarray data showed that the expression level of lncRNA *ENST00000508435* was significantly upregulated in breast cancer tissues, compared with that in paracancerous tissues. We performed RT-qPCR assays to measure the relative expression of *ENST00000508435* in 127 clinical breast cancer tissues (paired peritumor as control) and breast cancer cell lines (MCF10A versus BT549, MCF-7, MDA-MB-231 and T47D). *ENST00000508435* expressed significantly higher in both breast cancer tissues and cell lines (Figure 1(a,b)). Cellular fractionation assay shows that lncRNA *ENST00000508435* was mostly localized in the nuclei of breast cancer cells (Figure 1(c)).

### *ENST00000508435* is related to tumor size, lymph node metastasis and HER2

To characterize the role of *ENST00000508435* in breast cancers, we investigated the relationship between the expression of *ENST00000508435* and some typical clinical pathologic parameters [tumor size, breast cancer biomarkers (ER, PgR, and HER2), lymph node metastasis, ki67 and Nottingham grade]. We found that the expression of *ENST00000508435* was positively correlated with tumor size, lymph node metastasis and HER2 (Supplementary Table 1).

### Ectopic expression of *ENST00000508435* intensively promotes breast cancer cell migration

**Figure 2.**
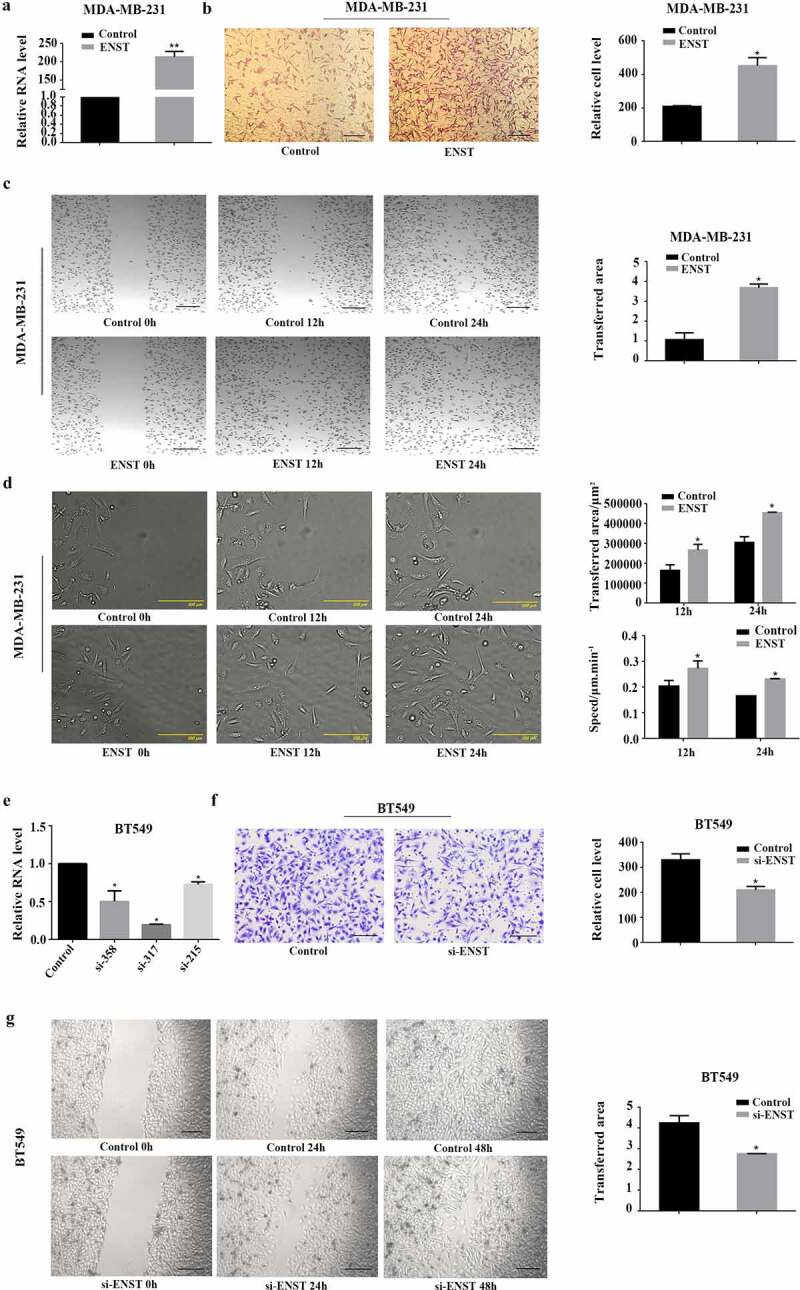
*ENST00000508435* (ENST for short) can promote migration of breast cancer cells. (a) RT-qPCR to detect the overexpression efficiency when pcDNA-*ENST00000508435* plasmid were transfected into MDA-MB-231 cells. (b-c) After overexpression for 48 h, transwell assays and cell wound healing were carried out to detect cell migration (left panel), the right panel are the statistical charts. Scale bar is 200 μm. (d) Cell migration was assessed by wound scratch assay in the following 24 h in MDA-MB-231. (e) RT-qPCR to detect inhibition efficiency when down-regulating *ENST00000508435* through siRNA in BT549 cells. siRNA-358, siRNA-317, siRNA-215 are three different interfering targets of *ENST00000508435*. (f-g) Cell migration detection when *ENST00000508435* was knocked down. left panel, cell migration; right panel, statistical charts. Scale bar is 200 μm. Data are shown as the mean ±S.D. based on five independent experiments. *P< 0.05 versus control; **P< 0.01 versus control

To assess the biological function of *ENST00000508435*, we examined the impact of *ENST00000508435* expression level on the migration of breast cancer cells with cell transwell assays and wound healing. The full length of pcDNA-*ENST00000508435* cDNA was transfected into breast cancer MDA-MB-231 cells by plasmid vectors. The expression was significantly increased about 200 times (Figure 2(a)). Transwell migration and wound healing assay showed that ectopic expression of *ENST00000508435* prominently increased the migration of MDA-MB-231 cells (Figure 2(b,c)). In addition, the movement of MDA-MB-231 cells was detected by JulI fluorescent cell analyzer (Figure 2(d)). Conversely, we performed reciprocal experiments to study the effects of knockdown on the migration in BT549 cells, which express high levels of *ENST00000508435*. Expression of *ENST00000508435* was notably down regulated upon specific siRNA transfection (Figure 2(e)). Furthermore, down-regulation of *ENST00000508435* expression impaired all migration capacity (Figure 2(f,g)).

### Association of *ENST00000508435* and Fragile X-Related 1 gene (FXR1)

**Figure 3.**
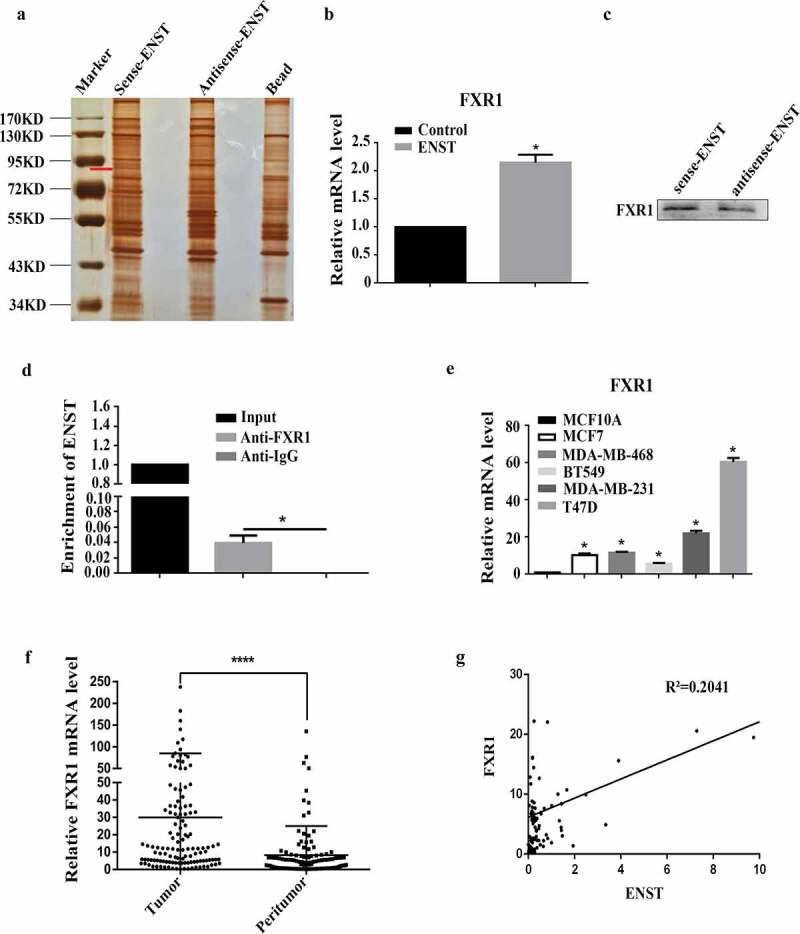
*ENST00000508435* (ENST for short) can bind to FXR1. (a) *ENST00000508435* specific combining proteins were detected by RNA pull down, and silver stain of the SDS-PAGE gel indicated the specific band was between 72 and 95 kDa as indicated by the red line, which was used for mass spectrometry. (b) After overexpression of *ENST00000508435* for 48 h in MDA-MB-231 cells, the mRNA level of FXR1 was higher than the control. (c) Western blot (BT549 cell lines) verified FXR1 can combine to biotinylated lncRNA *ENST00000508435*. (d) RIP experiments were executed using the FXR1 or IgG antibody to immunoprecipitate RNA and the purified RNA was used to detect *ENST00000508435* in BT549 cell, the enrichment of *ENST00000508435* was normalized to input. *P< 0.05 versus control; (e-f) RT-qPCR to detect the expression of FXR1 in five different breast cancer cell lines (e) (n = 5) and breast tissues (f). (g) The correlation analysis between *ENST00000508435* and FXR1 by SPSS. Data are shown as the mean ±S.D. (n = 5). *P< 0.05 versus control; ****P< 0.0001 versus control

LncRNAs exert important roles in regulating cell processes by binding with proteins. Recent research have reported that lncRNA HOTAIR can recruit PRC2 to their target genes [6,7]. Therefore, we examined whether *ENST00000508435* affects migration in a similar manner. At first, RNA pull-down assay was performed with biotin labeled *ENST00000508435* synthesized *in vitro*. Bands specific to *ENST00000508435* were excised for mass spectrometry (Figure 3(a)). In addition to nonspecific binding proteins, FXR1 had the highest unique peptides in all proteins (Supplementary Table 2). Then we performed qPCR to seek the specific band (Figure 3(b)), and western blot (protein captured from RNA pull-down assays in BT549 cells) to verify the mass spectrometry result. FXR1 enriched by sense RNA compared with antisense RNA (Figure 3(c)). We further used cell extracts from BT549 cell lines to perform RNA immunoprecipitation (RIP) with an antibody against FXR1. RT-qPCR studies showed that *ENST00000508435* was significantly enriched by the FXR1 antibody (Figure 3(d)). FXR1 expressed significantly higher in both breast cancer cell lines (MCF10A versus BT549, MCF7, MDA-MB-231, MDA-MB-468 and T47D) and tissues (Figure 3(e,f)). Furthermore, we found that *ENST00000508435* and FXR1 were positively correlated (Figure 3(g)).

### Knockdown of FXR1 inhibits the effects of *ENST00000508435* on cell migration

**Figure 4.**
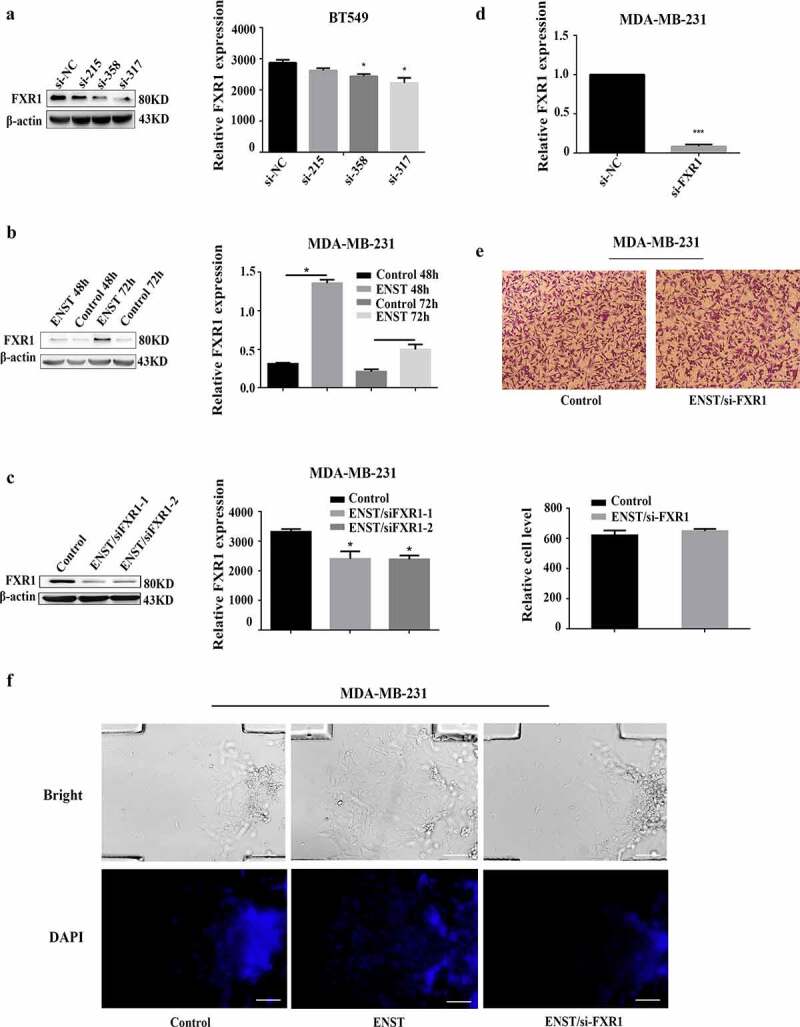
Knockdown of FXR1 abolished the effects of *ENST00000508435* (ENST for short) on cell migration. (a) BT549 cells were transfected with siRNA-*ENST00000508435*, western blot to detect the FXR1 expression. (b) MDA-MB-231 cells were transfected with pcDNA-*ENST00000508435*, western blot to detect the FXR1 expression in 48 h and 72 h. (c and d) Western blot or RT-qPCR to detect the transfection efficiency of FXR1 in MDA-MB-231 cells. (e) After transfection of pcDNA-*ENST00000508435* and siRNA-FXR1 for 48 h, transwell assays were carried out to detect cell migration of MDA-MB-231, scale bar is 200 μm. (f) Microfluidic chip invasion assay were carried out to detect cell invasion of MDA-MB-231. DAPI stained nucleus. Scale bar is 100 μm. Data show a representative of five independent experiments. Error bars indicate S.D. (n = 5). *P< 0.05 versus control; ***P< 0.001 versus control

We investigated the role of *ENST00000508435* on FXR1 function by ectopic expression of *ENST00000508435* in MDA-MB-231 or down-regulation *ENST00000508435* expression with ENST siRNA-homo-317 in MDA-MB-231 and BT549 cell lines. The expression of FXR1 was up-regulated or down-regulated when *ENST00000508435* was overexpressed or knocked down (Figure 4(a,b)), respectively. In cell migration assay (MDA-MB-231 cell lines), the expression of FXR1 was knocked down (verified by RT-qPCR and western blot assay after 48 h transfection) (Figure 4(c,d)), which inhibited the effects of *ENST00000508435* on cell migration and invasion through transwell (Figure 4(e)) and microfluidic chip invasion assay (Figure 4(f)). These findings suggested that FXR1 mediate the effect of *ENST00000508435*. We also analyzed the relationship between expression of FXR1 and clinical pathological parameters [tumor size, breast cancer biomarkers (ER, PgR and HER2), lymph node metastasis, ki67 and Nottingham grade], and found that the expression of FXR1 was positively correlated with lymph node metastasis (Supplementary Table 3).

### *ENST00000508435* promotes breast cancer lung metastasis

**Figure 5.**
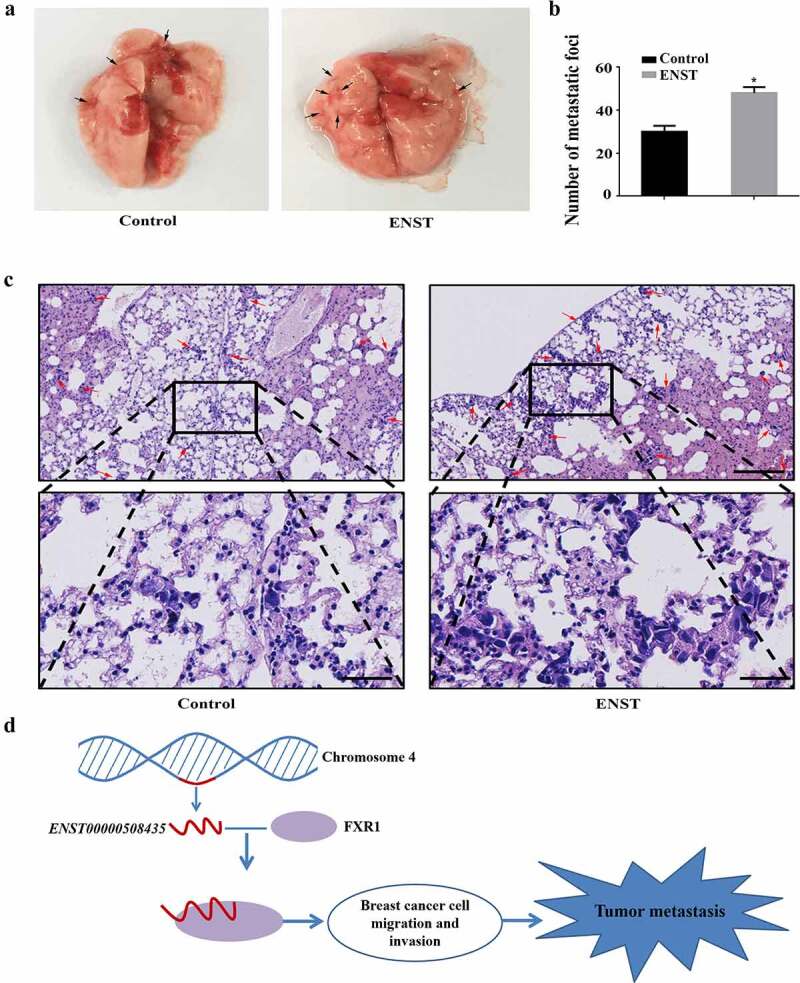
*ENST00000508435* (ENST for short) promotes breast cancer lung metastasis *in vivo*. (a) After 4 weeks of injection of *ENST00000508435* overexpression cells or control MDA-MB-231 cells by tail vein of BALB/c nude mice, bright-field imaging in the lungs were obtained (n = 8 for each). B-C, H&E staining (b) and average number of metastatic foci quantified from four H&E stained sections are graphed (c). Images of H&E staining were taken with 10× and 40× magnification. Arrows show the sites of metastasis. Scale bar are 200 μm and 50 μm, respectively. Data are shown as the mean ±S.D. (n = 5). *P< 0.05 versus control. (d) Schematic diagram of *ENST00000508435* promoting breast cancer metastasis and invasion via FXR1

To detect the *in vivo* metastatic ability of *ENST00000508435* in breast cancer cells, we injected MDA-MB-231 cells, stably expressing *ENST00000508435*, and control cells, respectively, into BALB/c nude mice via the tail vein to compare the abilities of these cell lines of forming lung metastatic nodules. Accordingly, the number of metastatic nodules on the lung surfaces (Figure 5(a)) and metastatic foci after H&E staining (Figure 5(b,c)) in the mice that were injected with *ENST00000508435-*overexpressed cells was obviously more than that within control groups.

## Discussion

The pathological development of breast cancer is related to the inactivation of tumor suppressor gene and abnormal molecular signaling pathway [8,9]. At the same time, the overexpression of oncogene is closely related to metastasis, recurrence, poor prognosis and short survival time. Benefit from the advancement of therapies (chemo, hormone, immuno and gene), long-term survival of breast cancer patients has become possible. However, up to 70% of lymph node positive patients tend to develop distant metastases, which is quite detrimental [10]. Early diagnosis biomarkers for breast cancer are mainly concentrated on protein coding genes, such as TBX2/3, Ki-67, HER-2, ER, VEGF and PR, among which HER2 protein expression is acknowledgedly related to metastasis and survival time. Thus, HER2 protein was chosen to judge the prognosis of breast cancer [11,12]. Compared with the protein coding genes, lncRNAs is relatively massive [11,12]. Recently, lncRNAs have been proved playing a critical role in tumorigenesis, and contributing to a diverse of biological functions in human cancers [13,14]. For example, HOTAIR is an early reported lncRNA, which regulates genes by chromosome remodeling [15]. Increased expression of HOTAIR in primary and metastatic breast cancer, especially of highly metastatic patients, is a significant predictor of subsequent metastasis and death [16]. In the current study, we found that the expression of lncRNA *ENST0000508435*, a novel transcript on chromosome 4, was greatly upregulated in breast cancer tissues and positively correlated with tumor size, lymph node metastasis and HER2 expression. This result suggests that *ENST00000508435* may be involved in the pathogenesis of breast cancer.

Migration ability is a typical biological behavior of malignant tumor cells. More and more evidence reportedly support lncRNA as a prominent factor of regulating cancer cell migration and invasion. For example, the expression of LSINCT5 in primary breast cancer tissues is ten times higher than that in normal tissues, which enhances the invasion of breast cancer cells via chemokine receptor (CXCR4) [17]. In addition, lncRNA-MALAT1 and ZXF1 promoted the migration and invasion of lung cancer cells [17]. To investigate the biological role of *ENST00000508435* in breast cancer, we assessed cell migration in both BT549 and MDA-MB-231 cells with transwell, wound healing and microfluidic chip invasion assay, and we found that *ENST00000508435* remarkably promoted breast cancer cell migration. However, the detailed mechanism remains to be explored.

It is been reported that lncRNAs may impact on cellular functions through various mechanisms, such as protein-protein scaffolds, protein-DNA, protein decoys, miRNA sponges, and regulators of translation [13]. For instance, lncRNA Xist was responsible for recruiting PRC2 to specific genomic regions, and EZH2 served as the RNA-binding subunit [13]. In addition, the conserved A-repeat domain of lncRNA Xist could be identified by PRC2 complex protein EZH2 [18–20]. HOX cluster-derived lncRNA, HOTAIR, was validated to recruit PRC2 to its target gene [6,21]. Therefore, we hypothesized that *ENST00000508435* was also regulated by related binding proteins. Performing RNA pull-down assay, mass spectrometry, western blot, and RIP, we identified that Fragile X-Related 1 gene (FXR1) was the specific binding protein of *ENST00000508435*.

FXR1 belongs to the RNA-binding proteins family, including Fragile X mental retardation-related gene 1 (FMR1) and Fragile X-related gene 2 (FXR2) [6,21]. RNA binding proteins (RBPs) are important molecules in the process of RNA metabolism, from synthesis to degradation. RBPs regulate RNA metabolism through RNA-protein and protein–protein interaction networks [22]. FXR1 combines with RNA and is involved in special diseases via RNA [23]. Jorge Bolivar showed us that FXR1 was involved in many biological processes of miRNA, which is essential for the maturation of miR-1, miR-124 and miR-9 [24]. A recent observation indicated lncRNA TUG1 regulates ApoM to promote atherosclerosis progression through miR-92a/FXR1 axis [24]. Additionally, Qihong Huang identified a novel ribonucleprotein (RNP) complex (hnRNPK, FXR1, FXR2, PUF60, SF3B3), which is required for translational regulation of lncRNA to cause tumor invasion and metastasis [25]. In our study, overexpression of *ENST00000508435* significantly increased migration of MDA-MB-231 cells, which was inhibited by depletion of FXR1, supporting that the function of *ENST00000508435* in breast cancer migration depends on FXR1. FXR1 may act as a target of *ENST00000508435*.

What we have done here indicated that *ENST00000508435* participated in promoting migration of breast cancer by interacting with FXR1 (Figure 5(d)). Future investigations are needed to explore whether lncRNAs and RNA-binding proteins might serve as potential diagnostic and prognostic markers, or even novel therapeutic targets.

## Conclusion

LncRNA takes a great part in tumor progression, and its definite role in breast cancer, a complicated disease with a high mortality rate, remains to be revealed. Here, our work shows that lncRNA *ENST00000508435* significantly promotes breast cancer metastasis, and we initially report that the effect of *ENST00000508435* is mediated by FXR1, which is co-expressed and positively correlated with *ENST00000508435* in breast cancer cell lines and tissues. To sum up, *ENST00000508435* and FXR1 may represent new biomarkers and potential targets for breast cancer clinical therapy.

## Supplementary Material

Supplemental Material

## Abbreviations

lncRNA,: long non-coding RNA;
RIP,: RNA immunoprecipitation;
ER,: estrogen receptor;
PgR,: progesterone receptor;
HER2,: human epidermal growth factor receptor 2;
PBS,: phosphate-buffered saline;
RT-qPCR,: Real-time quantitative PCR;
FXR1,: Fragile X medntal retardation 1;
XIST,: X-inactive-specific transcript;
EZH2,: enhancer of zeste homolog 2;
min,: minute;
h,: hour.

## References

[1] Sung H, Ferlay J, Siegel RL, et al. Global cancer statistics 2020: GLOBOCAN estimates of incidence and mortality worldwide for 36 cancers in 185 countries. CA Cancer J Clin. 2021. DOI:10.3322/caac.21660 33538338

[2] Prensner JR, Chinnaiyan AM. The emergence of lncRNAs in cancer biology. Cancer Discov. 2011;1:391. 22096659 10.1158/2159-8290.CD-11-0209 PMC3215093

[3] Ma L, Bajic VB, Zhang Z. On the classification of long non-coding RNAs. Rna Biol. 2013;10:925–933. 23696037 10.4161/rna.24604 PMC4111732

[4] Bartonicek N, Maag JL, Dinger ME. Long noncoding RNAs in cancer: mechanisms of action and technological advancements. Mol Cancer. 2016;15:43. 27233618 10.1186/s12943-016-0530-6 PMC4884374

[5] Liao X-H, Wang J-G, Li L-Y, et al. Long intergenic non-coding RNA APOC1P1-3 inhibits apoptosis by decreasing α-tubulin acetylation in breast cancer. Cell Death Dis. 2016;7(5):e2236. 27228351 10.1038/cddis.2016.142 PMC4917671

[6] Chu C, Qu K, Zhong FL, et al. Genomic maps of long noncoding RNA occupancy reveal principles of RNA-chromatin interactions. Mol Cell. 2011;44:667–678. 21963238 10.1016/j.molcel.2011.08.027 PMC3249421

[7] Rinn JL, Kertesz M, Wang JK, et al. Functional demarcation of active and silent chromatin domains in human HOX loci by noncoding RNAs. Cell. 2007;129:1311–1323. 17604720 10.1016/j.cell.2007.05.022 PMC2084369

[8] Pagani O, Senkus E, Wood W, et al. International guidelines for management of metastatic breast cancer: can metastatic breast cancer be cured? J Natl Cancer Inst. 2010;102:456–463. 20220104 10.1093/jnci/djq029 PMC3298957

[9] Stevens RG, Brainard GC, Blask DE, et al. Breast cancer and circadian disruption from electric lighting in the modern world. CA Cancer J Clin. 2014;64:207–218. 24604162 10.3322/caac.21218 PMC4038658

[10] Desantis C, Ma J, Bryan L, et al. Breast cancer statistics, 2013. Ca A Cancer J Clinicians. 2014;64:52. 10.3322/caac.21203 24114568

[11] Penault-Llorca F, Radosevic-Robin N. Biomarkers of residual disease after neoadjuvant therapy for breast cancer. Nat Rev Clin Oncol. 2016;13:487–503. 26856744 10.1038/nrclinonc.2016.1

[12] Nayar U, Cohen O, Kapstad C, et al. Acquired HER2 mutations in ER(+) metastatic breast cancer confer resistance to estrogen receptor-directed therapies. Nat Genet. 2019;51:207–216. 30531871 10.1038/s41588-018-0287-5

[13] Kopp F, Mendell JT. Functional classification and experimental dissection of long noncoding RNAs. Cell. 2018;172:393–407. 29373828 10.1016/j.cell.2018.01.011 PMC5978744

[14] Grelet S, Link LA, Howley B, et al. A regulated PNUTS mRNA to lncRNA splice switch mediates EMT and tumour progression. Nat Cell Biol. 2017;19:1105–1115. 28825698 10.1038/ncb3595 PMC5578890

[15] Gupta RA, Shah N, Wang KC, et al. Long non-coding RNA HOTAIR reprograms chromatin state to promote cancer metastasis. Nature. 2010;464:1071–1076. 20393566 10.1038/nature08975 PMC3049919

[16] Tao D, Zhang Z, Liu X, et al. LncRNA HOTAIR promotes the invasion and metastasis of oral squamous cell carcinoma through metastasis-associated gene 2. Mol Carcinog. 2020;59:353–364. 31995261 10.1002/mc.23159

[17] Silva JM, Boczek NJ, Berres MW, et al. LSINCT5 is over expressed in breast and ovarian cancer and affects cellular proliferation. RNA Biology. 2011;8(3):496. 21532345 10.4161/rna.8.3.14800

[18] Kaneko S, Li G, Son J, et al. Phosphorylation of the PRC2 component Ezh2 is cell cycle-regulated and up-regulates its binding to ncRNA. Genes Dev. 2010;24:2615–2620. 21123648 10.1101/gad.1983810 PMC2994035

[19] Maenner S, Blaud M, Fouillen L, et al. 2-D structure of the A region of Xist RNA and its implication for PRC2 association. Plos Biol. 2010;8:e1000276. 20052282 10.1371/journal.pbio.1000276 PMC2796953

[20] Kanhere A, Viiri K, Araujo CC, et al. Short RNAs are transcribed from repressed polycomb target genes and interact with polycomb repressive complex-2. Mol Cell. 2010;38:675–688. 20542000 10.1016/j.molcel.2010.03.019 PMC2886029

[21] Imai-Sumida M, Dasgupta P, Kulkarni P, et al. Genistein represses HOTAIR/chromatin remodeling pathways to suppress kidney cancer. Cell Physiol Biochem. 2020;54:53–70. 31961100 10.33594/000000205 PMC7382546

[22] Gerstberger S, Hafner M, Tuschl T. A census of human RNA-binding proteins. Nat Rev Genet. 2014;15:829–845. 25365966 10.1038/nrg3813 PMC11148870

[23] Herman AB, Vrakas CN, Ray M, et al. FXR1 is an IL-19-responsive RNA-binding protein that destabilizes pro-inflammatory transcripts in vascular smooth muscle cells. Cell Rep. 2018;24:1176–1189. 30067974 10.1016/j.celrep.2018.07.002 PMC11004729

[24] Xu XL, Zong R, Li Z, et al. FXR1P but not FMRP regulates the levels of mammalian brain-specific microRNA-9 and microRNA-124. J Neurosci. 2011;31:13705–13709. 21957233 10.1523/JNEUROSCI.2827-11.2011 PMC3446782

[25] Gumireddy K, Li A, Yan J, et al. Identification of a long non-coding RNA-associated RNP complex regulating metastasis at the translational step. Embo J. 2013;32:2672–2684. 23974796 10.1038/emboj.2013.188 PMC3801433

