# Supplementary material for: Long noncoding RNA ENST00000508435 promotes migration of breast cancer via FXR 1
Source: Cell Adh Migr. 2021 May 30;15(1):140–51. doi: 10.1080/19336918.2021.1921402 (PMC8168597; doi:10.1080/19336918.2021.1921402)
Supplement: Supplemental Material [file KCAM_A_1921402_SM2473.zip › Document.rtf]

Supplementary Table 1�DRelationship between ENST00000508435 expressions in cancer tissues and clinicopathologic parameters (n=127)
Supplementary Table 2: Mass spectrometry experiments were performed to select the target gene.
Supplementary Table 3�DRelationship between FXR1 expressions in cancer tissues and clinicopathologic parameters (n=127)
