# Supplementary material for: Long noncoding RNA ENST00000508435 promotes migration of breast cancer via FXR 1
Source: Cell Adh Migr. 2021 May 30;15(1):140–51. doi: 10.1080/19336918.2021.1921402 (PMC8168597; doi:10.1080/19336918.2021.1921402)
Supplement: Supplemental Material [file KCAM_A_1921402_SM2473.zip › Supplementary material_CAM_revised_CLEAN.doc]

**Supplementary Table 1．Relationship between *ENST00000508435* expressions in cancer tissues and clinicopathologic parameters (n=127**)

|  | n | ***ENST00000508435* expression** | | *P* value |
| --- | --- | --- | --- | --- |
|  |  | High | Low |  |
| **Tumor size (cm)** |  |  |  | 0.0428* |
| ≥2.5 | 76 | 60 | 16 |  |
| <2.5 | 51 | 31 | 20 |  |
| **Lymph node metastasi**s |  |  |  | 0.0123* |
| Yes | 63 | 52 | 11 |  |
| No | 64 | 39 | 25 |  |
| **Nottingham grade** |  |  |  | 0.2788 |
| Grade I | 66 | 51 | 15 |  |
| Grade II | 47 | 30 | 17 |  |
| Grade III | 14 | 10 | 4 |  |
| **ER** |  |  |  | 0.8952 |
| ≥ 1% | 77 | 56 | 21 |  |
| < 1% | 50 | 35 | 15 |  |
| **PgR** |  |  |  | 0.2258 |
| ≥ 1% | 62 | 48 | 14 |  |
| < 1% | 65 | 43 | 22 |  |
| **HER2#** |  |  |  | 0.014* |
| Overexpression | 59 | 49 | 10 |  |
| Negative | 68 | 42 | 26 |  |
| **ki67** |  |  |  | 0.9985 |
| ≥ 20% | 97 | 69 | 28 |  |
| < 20% | 30 | 22 | 8 |  |

*The *ENST00000508435* expression is statistically significant, *P*<0.05 vs control.

\# HER2 status was validated by fluorescence in situ hybridization.

**Supplementary Table 2:** **Mass spectrometry experiments were performed to select the target gene.**

| Accession | Description | Score | Coverage | # Proteins | # Unique Peptides | # Peptides | # PSMs | # AAs | MW [kDa] | calc. pI |
| --- | --- | --- | --- | --- | --- | --- | --- | --- | --- | --- |
| **P12883** | **Myosin-7 OS=Homo sapiens GN=MYH7 PE=1 SV=5 - [MYH7_HUMAN]** | **573.87** | **3.10** | **6** | **4** | **4** | **9** | **1935** | **223.0** | **5.80** |
| P35527 | Keratin, type I cytoskeletal 9 OS=Homo sapiens GN=KRT9 PE=1 SV=3 - [K1C9_HUMAN] | 458.67 | 10.43 | 1 | 6 | 6 | 11 | 623 | 62.0 | 5.24 |
| P04264 | Keratin, type II cytoskeletal 1 OS=Homo sapiens GN=KRT1 PE=1 SV=6 - [K2C1_HUMAN] | 399.58 | 13.66 | 2 | 9 | 11 | 13 | 644 | 66.0 | 8.12 |
| P13645 | Keratin, type I cytoskeletal 10 OS=Homo sapiens GN=KRT10 PE=1 SV=6 - [K1C10_HUMAN] | 369.34 | 12.33 | 20 | 7 | 7 | 8 | 584 | 58.8 | 5.21 |
| **P51114#** | **Fragile X mental retardation syndrome-related protein 1 OS=Homo sapiens GN=FXR1 PE=1 SV=3 - [FXR1_HUMAN]** | **308.92** | **10.31** | **2** | **7** | **8** | **10** | **621** | **69.7** | **6.15** |
| **O43390** | **Heterogeneous nuclear ribonucleoprotein R OS=Homo sapiens GN=HNRNPR PE=1 SV=1 - [HNRPR_HUMAN]** | **288.65** | **9.79** | **2** | **6** | **6** | **10** | **633** | **70.9** | **8.13** |
| P17661 | Desmin OS=Homo sapiens GN=DES PE=1 SV=3 - [DESM_HUMAN] | 265.96 | 3.19 | 1 | 1 | 1 | 4 | 470 | 53.5 | 5.27 |
| P35908 | Keratin, type II cytoskeletal 2 epidermal OS=Homo sapiens GN=KRT2 PE=1 SV=2 - [K22E_HUMAN] | 225.54 | 8.14 | 5 | 3 | 5 | 5 | 639 | 65.4 | 8.00 |
| Q06787 | Fragile X mental retardation protein 1 OS=Homo sapiens GN=FMR1 PE=1 SV=1 - [FMR1_HUMAN] | 216.47 | 8.54 | 1 | 6 | 7 | 8 | 632 | 71.1 | 7.42 |
| Q7Z417 | Nuclear fragile X mental retardation-interacting protein 2 OS=Homo sapiens GN=NUFIP2 PE=1 SV=1 - [NUFP2_HUMAN] | 170.87 | 4.32 | 1 | 2 | 2 | 2 | 695 | 76.1 | 8.70 |
| **Q00839** | **Heterogeneous nuclear ribonucleoprotein U OS=Homo sapiens GN=HNRNPU PE=1 SV=6 - [HNRPU_HUMAN]** | **159.04** | **5.09** | **1** | **5** | **5** | **5** | **825** | **90.5** | **6.00** |
| P07900 | Heat shock protein HSP 90-alpha OS=Homo sapiens GN=HSP90AA1 PE=1 SV=5 - [HS90A_HUMAN] | 90.92 | 2.73 | 4 | 2 | 2 | 2 | 732 | 84.6 | 5.02 |
| P13647 | Keratin, type II cytoskeletal 5 OS=Homo sapiens GN=KRT5 PE=1 SV=3 - [K2C5_HUMAN] | 85.22 | 3.05 | 1 | 2 | 2 | 2 | 590 | 62.3 | 7.74 |
| P06732 | Creatine kinase M-type OS=Homo sapiens GN=CKM PE=1 SV=2 - [KCRM_HUMAN] | 83.84 | 5.77 | 1 | 1 | 1 | 2 | 381 | 43.1 | 7.25 |
| **P11498** | **Pyruvate carboxylase, mitochondrial OS=Homo sapiens GN=PC PE=1 SV=2 - [PYC_HUMAN]** | **82.94** | **2.46** | **1** | **3** | **3** | **3** | **1178** | **129.6** | **6.84** |
| Q5SSJ5 | Heterochromatin protein 1-binding protein 3 OS=Homo sapiens GN=HP1BP3 PE=1 SV=1 - [HP1B3_HUMAN] | 82.51 | 3.98 | 1 | 2 | 2 | 2 | 553 | 61.2 | 9.67 |
| P60709 | Actin, cytoplasmic 1 OS=Homo sapiens GN=ACTB PE=1 SV=1 - [ACTB_HUMAN] | 72.18 | 2.67 | 10 | 1 | 1 | 1 | 375 | 41.7 | 5.48 |
| P19338 | Nucleolin OS=Homo sapiens GN=NCL PE=1 SV=3 - [NUCL_HUMAN] | 60.65 | 2.25 | 1 | 2 | 2 | 2 | 710 | 76.6 | 4.70 |
| P02786 | Transferrin receptor protein 1 OS=Homo sapiens GN=TFRC PE=1 SV=2 - [TFR1_HUMAN] | 59.15 | 1.32 | 1 | 1 | 1 | 1 | 760 | 84.8 | 6.61 |
| Q02809 | Procollagen-lysine,2-oxoglutarate 5-dioxygenase 1 OS=Homo sapiens GN=PLOD1 PE=1 SV=2 - [PLOD1_HUMAN] | 53.76 | 0.96 | 1 | 1 | 1 | 1 | 727 | 83.5 | 6.95 |
| Q9NR30 | Nucleolar RNA helicase 2 OS=Homo sapiens GN=DDX21 PE=1 SV=5 - [DDX21_HUMAN] | 51.84 | 1.15 | 1 | 1 | 1 | 1 | 783 | 87.3 | 9.28 |
| P08237 | 6-phosphofructokinase, muscle type OS=Homo sapiens GN=PFKM PE=1 SV=2 - [K6PF_HUMAN] | 38.51 | 1.15 | 3 | 1 | 1 | 1 | 780 | 85.1 | 7.99 |
| Q9Y4W6 | AFG3-like protein 2 OS=Homo sapiens GN=AFG3L2 PE=1 SV=2 - [AFG32_HUMAN] | 38.19 | 1.51 | 1 | 1 | 1 | 1 | 797 | 88.5 | 8.66 |
| P26639 | Threonine--tRNA ligase, cytoplasmic OS=Homo sapiens GN=TARS PE=1 SV=3 - [SYTC_HUMAN] | 34.09 | 1.11 | 2 | 1 | 1 | 1 | 723 | 83.4 | 6.67 |
| P16070 | CD44 antigen OS=Homo sapiens GN=CD44 PE=1 SV=3 - [CD44_HUMAN] | 31.84 | 1.08 | 1 | 1 | 1 | 1 | 742 | 81.5 | 5.33 |
| O95399 | Urotensin-2 OS=Homo sapiens GN=UTS2 PE=1 SV=1 - [UTS2_HUMAN] | 30.26 | 6.45 | 2 | 1 | 1 | 1 | 124 | 14.3 | 7.80 |
| P02768 | Serum albumin OS=Homo sapiens GN=ALB PE=1 SV=2 - [ALBU_HUMAN] | 29.07 | 1.15 | 1 | 1 | 1 | 1 | 609 | 69.3 | 6.28 |
| Q7L2J0 | 7SK snRNA methylphosphate capping enzyme OS=Homo sapiens GN=MEPCE PE=1 SV=1 - [MEPCE_HUMAN] | 28.72 | 1.16 | 1 | 1 | 1 | 1 | 689 | 74.3 | 9.57 |
| O75534 | Cold shock domain-containing protein E1 OS=Homo sapiens GN=CSDE1 PE=1 SV=2 - [CSDE1_HUMAN] | 24.42 | 1.13 | 1 | 1 | 1 | 1 | 798 | 88.8 | 6.25 |

**#** We selected from the higher peptide score of several target gene from three mass spectrometry experiments. Then we use qPCR to verify the expression of the gene, and finally select FXR1 as the target gene.

**Supplementary Table 3．Relationship between *FXR1* expressions in cancer tissues and clinicopathologic parameters (n=127)**

|  | n | ***FXR1* expression**^a^ | | *P* value |
| --- | --- | --- | --- | --- |
|  |  | High | Low |  |
| **Tumor size (cm)** |  |  |  | 0.3866 |
| ≥2.5 | 76 | 57 | 19 |  |
| <2.5 | 51 | 34 | 17 |  |
| **Lymph node metastasi**s |  |  |  | 0.0349^*^ |
| Yes | 63 | 51 | 12 |  |
| No | 64 | 40 | 24 |  |
| **Nottingham grade** |  |  |  | 0.2235 |
| Grade I | 66 | 49 | 17 |  |
| Grade II | 47 | 30 | 17 |  |
| Grade III | 14 | 12 | 2 |  |
| **ER** |  |  |  | 0.3685 |
| ≥ 1% | 77 | 56 | 21 |  |
| < 1% | 50 | 35 | 15 |  |
| **PgR** |  |  |  | 0.672 |
| ≥ 1% | 62 | 46 | 16 |  |
| < 1% | 65 | 45 | 20 |  |
| **HER2^#^** |  |  |  | 0.9294 |
| Overexpression | 59 | 42 | 17 |  |
| Negative | 68 | 49 | 19 |  |
| **ki67** |  |  |  | 0.6417 |
| ≥ 20% | 97 | 68 | 29 |  |
| < 20% | 30 | 23 | 7 |  |

a The cutoff value is the median expression level of *FXR1*.

* The *FXR1* expression is statistically significant, *P*<0.05 vs control.

\# HER2 status was validated by fluorescence in situ hybridization.
